# Dancing

**Published:** 1871-03

**Authors:** 


### DANCING

WE are in hearty accord at present with the official expression of sentiment of the Roman Catholic Church, that public, promiscuous dancing is pernicious to the highest, purest and best interests of social life. The Baltimore *Episcopal Methodist*, in reference to a decision of the council of Roman Catholic bishops on the subject, says, " The opinion of this council derives great weight from the fact that it is based upon the information obtained in the confessional from one end of the country to the other. The effect of these dances upon the mind and morals with us is an inference; with them it is a disclosure; and we do not know how its weight is to be resisted by the votaries of the fashion."

Some reader may exclaim, in a kind of lofty contempt, " Fudge!" but we must take human nature as it is, and from the stand-point of a daughter's purity, thoughtful parents are invited to consider the subject, for it is one of great social importance.

